# Takotsubo cardiomyopathy Afatinib-related in a non-small cell lung cancer patient: Case report

**DOI:** 10.3389/fcvm.2022.1060813

**Published:** 2022-11-22

**Authors:** German E. Ramos, Christian Caglevic, Juan F. Bulnes, Sergio E. Panay, Mario I. Zapata, Andrés J. Daniele, Manuel E. Rodríguez

**Affiliations:** ^1^Department of Cardiology, Instituto Oncológico Fundación Arturo López Pérez, Santiago, Chile; ^2^Ehocardiography Laboratory, Instituto Oncológico Fundación Arturo López Pérez, Santiago, Chile; ^3^Department of Cancer Research, Instituto Oncológico Fundación Arturo López Pérez, Santiago, Chile; ^4^Clinical Trials Unit, Instituto Oncológico Fundación Arturo López Pérez, Santiago, Chile; ^5^Department of Cardiology, Pontificia Universidad Católica de Chile, Santiago, Chile; ^6^Department of Oncology, Instituto Oncológico Fundación Arturo López Pérez, Santiago, Chile; ^7^Department of Cardio-Oncology, Institute of Oncology “Angel H. Roffo”, Buenos Aires, Argentina

**Keywords:** Takotsubo (stress) cardiomyopathy, Afatinib, EGFR mutation, tyrosine kinase inhibition, NSCLC

## Abstract

Endothelial Growth Factor Receptor (EGFR) mutations are frequently found among NSCLC patients. Second-generation Tyrosine Kinase Inhibitor (TKI) Afatinib is frequently used in this population of patients achieving better results than cytotoxic chemotherapy in terms of survival and progression. Afatinib-related cardiotoxicity has been rarely reported. Here we comment on a clinical case of a Takotsubo Cardiomyopathy Afatinib-induced in an NSCLC patient.

## Clinical case

A 57-year-old woman with heavy active smoking history was hospitalized due to convulsive syndrome. A brain computed tomography (CT) scan showed an expansive lesion suggesting metastasis. A thorough workup in search of a primary malignancy including a thoracic CT scan reported a mass in the right lung upper lobe. Lung biopsy showed a squamous non-small cell lung cancer (NSCLC) with an Epidermal Growth Factor Receptor (EGFR) exon 19 deletion. The patient started treatment with Afatinib continuously, achieving partial clinical response according to Response Evaluation Criteria in Solid Tumors (RECIST) 1.1. Since the earliest cycles of Afatinib, the patient developed a mild skin rash. This situation is commonly observed among patients that are undergoing anti-EGFR treatments.

**FIGURE 1:**
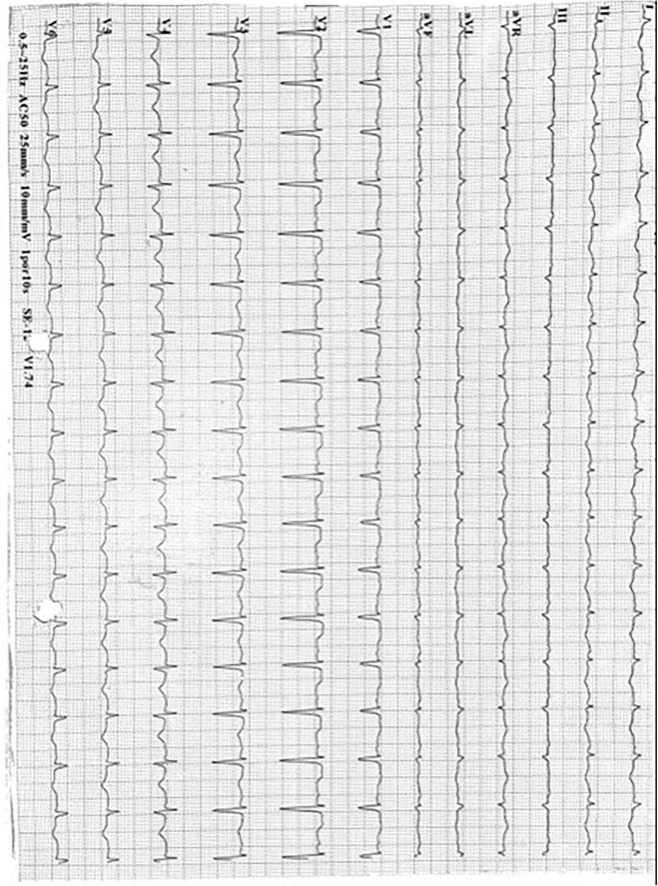
Electrocardiogram series. Shows the electrocardiogram at TTS diagnosis ST segment elevation in anterior and septal walls (V2 and V3), with symmetrical T wave inversion in all anterior segments (DI, aVL, v4 to V6), and prolonged corrected QT interval (more than 500 ms).

**FIGURE 2:**
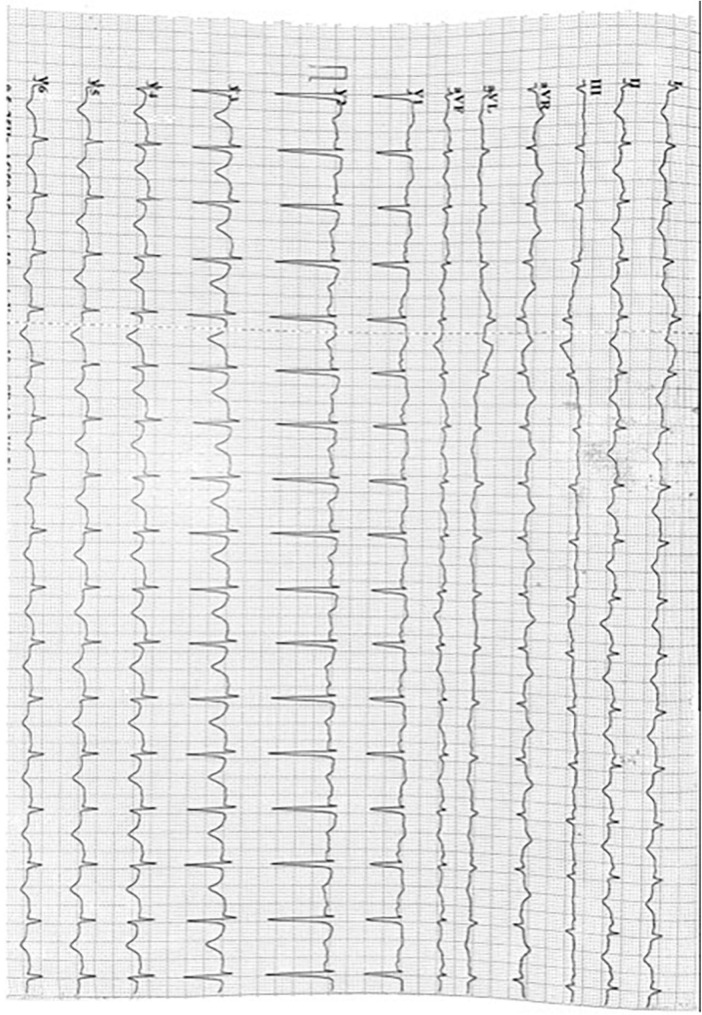
Electrocardiogram series. Shows electrocardiogram at day 2 since TTS diagnosis: A slight ST segment elevation (1 mm) in V1 is added compared with previous EKG.

**FIGURE 3:**
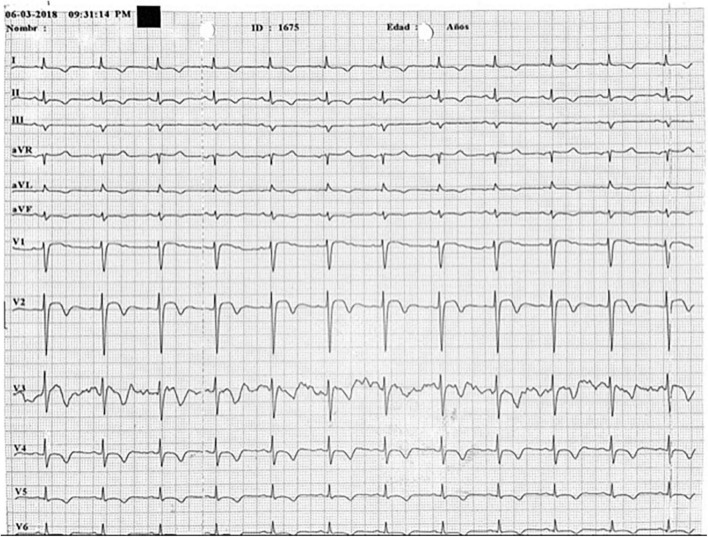
Electrocardiogram series. Shows electrocardiogram at day 7 since TTS diagnosis: A Wellens Pattern (type 1) is now observed in V2 and V3 leads t wave inversions and prolonged QT interval remains.

**FIGURE 4:**
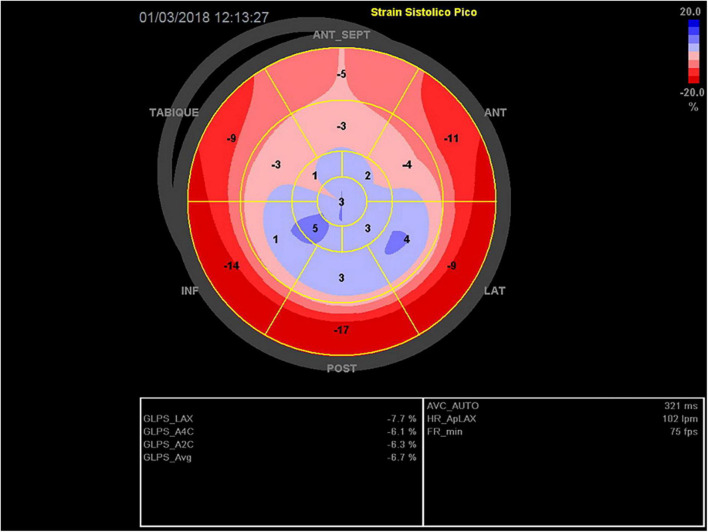
Global longitudinal strain (Bull’s eye plot) at day 0. Akinesia of all middle and apical segments, with circumferential pattern. Global systolic function is severely compromised (GLS −7%).

**FIGURE 5:**
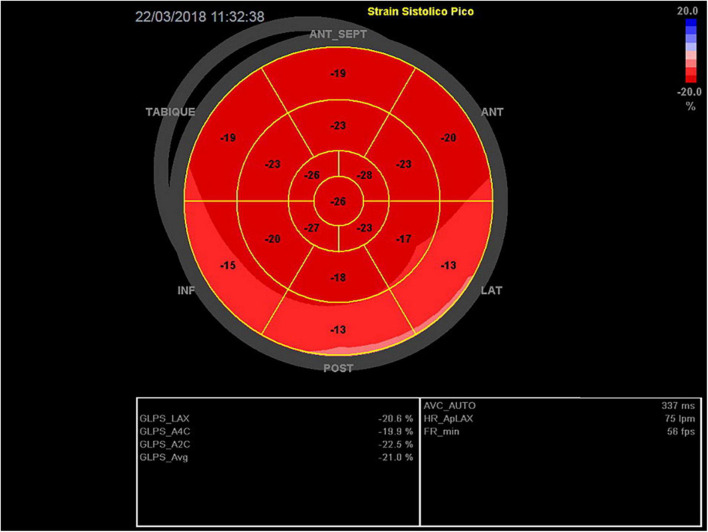
Global longitudinal strain (Bull’s eye plot) at day 22. There is a remarkable, and practically complete, recovery from the severe initial compromise of the segmentary wall motion abnormalities observed on day 0. Global systolic function is preserved (GLS −21%).

After 19 four-week cycles of Afatinib treatment, the patient was admitted due to a severe skin eruption that was considered secondary to Afatinib use, grade 3 according to CTCAE V5.0 (1). The dermatologist’s prescription indicated systemic steroidal therapy and, a multidisciplinary team confirmed that this severe cutaneous toxicity was related to Afatinib use, defining the interruption of this treatment, and achieving then the remission of the cutaneous syndrome. However, one week later after the hospitalization, the patient developed a rapid onset of upper extremity edema and dyspnea on minimal exertion. A thoracic CT showed progression of disease with a new mediastinal mass and superior vena cava thrombosis. Anticoagulation and a course of corticosteroids was started, with a good initial clinical response. By day 11 of hospitalization she presented an anxiety crisis followed by oppressive chest pain and dyspnea. Physical examination revealed tachycardia of 115 bpm, blood pressure 140/80 mm Hg, and oxygen saturation of 90% without oxygen support. The patient appeared to be in mild respiratory distress, with jugular vein distention, S3 gallop on cardiac auscultation, and crackles in both lower lung fields. Electrocardiogram showed ST segment elevation in anterior and septal walls (V2 and V3), with symmetrical T wave inversion in all anterior segments (DI, aVL, v4 to V6), and prolonged corrected QT interval (more than 500 ms). Electrocardiograms at TTS diagnosis, at day 2, and day 7 are shown in Figures 1–3, respectively. High-sensitivity troponin levels were raised (0.147 ng/mL, upper reference limit <0.03 ng/ml). A transthoracic echocardiogram (TTE) was performed, revealing akinesis of all middle and apical segments, basal anteroseptal hypokinesis and severe left ventricular systolic dysfunction with left ventricular ejection fraction (LVEF) of 30% (biplane Simpson method). Global longitudinal strain (GLS) – by speckle tracking technique – was severely reduced (−6.7%), displaying a circumferential pattern in the Bull’s eye plot (Figure 4). These findings were highly suggestive of Takotsubo cardiomyopathy syndrome (TTS). Facing a poor clinical scenario due to a NSCLC metastatic disease, multidisciplinary team (oncologist, intensive care unit and cardiologist) considered that undergoing a coronary angiographic study was an extraordinary measure, so it was discarded. Nonetheless, patient was transferred to the intensive care unit. Medical therapy was initiated, including vasodilators and diuretics, and beta-blockers were introduced after relieving pulmonary congestion. The patient evolved with a prompt clinical and biochemical recovery. Echocardiographic follow up showed progressive improvement of initial findings, achieving complete recovery at three weeks after diagnosis, with no segmental motion abnormalities, a LVEF of 60%, and a peak GLS of −21% (Figure 5). A liquid biopsy showed the presence of the T790M resistance mutation, and Osimertinib was started. Unfortunately, the patient died a few months later due to progression of disease.

## Discussion

Epidermal Growth Factor Receptors (EGFR) superfamily includes 4 subtypes of receptors currently named EGFR/ERbB1, ErbB2, ErbB3, and ERBb4. These tyrosine kinase EGFR groups have an important role in both embryological and postnatal development, including the cardiovascular system.

Endothelial Growth Factor Receptor (EGFR) amplification and mutations are involved in carcinogenesis, and among non-small cell lung cancer (NSCLC) patients show a high heterogeneity rate. Afatinib, a second-generation tyrosine kinase inhibitor (TKI), produces an irreversible covalent binding not only to EGFR but also to ErbB2, ErbB3, and ErbB4, achieving better results compared with gefitinib and cisplatin-doublet-based chemotherapy in NSCLC – EGFR mutated patients (2). Other EGFR-targeted treatments such as Gefitinib, Erlotinib, Cetuximab, and Panitumumab carry a very low risk of cardiotoxicity (3) and in the specific case of Afatinib-related cardiotoxicity mainly systolic dysfunction has only been sporadically reported (4).

The mechanism by which Afatinib may be involved with the development of cardiotoxicity is related to the “On/Off target” hypothesis. Tumor growth participates in the regulation of survival of cardiomyocytes by two different mechanisms that favor the development of cardiotoxicity. In the “on-target” mechanism the pharmacological target for the tyrosine kinase is similar in cardiomyocytes and in cancer cells and is involved in both proliferation and survival during tumorigenesis. In the “off-target” mechanism the TKI blocks a pathway or a specific kinase different from the original pharmacological target, carrying a risk of toxicity that depends on the inhibited pathway (5). Human epidermal growth factor receptor 2 (HER2) targeted treatment, such as Trastuzumab, triggers systolic dysfunction in approximately 10% of breast cancer patients that are exposed to this treatment (6). The mechanism of action of Afatinib includes not only inhibiting irreversibly both EGFR and HER 2 receptors. Based on the “on/off target mechanism,” if saturation of the EGFR receptor occurs (clinically expressed in our patient as severe skin toxicity), afatinib would additionally inhibit HER 2 receptors, generating cardiotoxicity through a mechanism similar to Trastuzumab and other anti HER2 monoclonal antibodies.

Takotsubo syndrome (TTS) was first reported by Sato in 1991 (7). It consists of an acute and transient systolic dysfunction, affecting mainly postmenopausal women, and often preceded by a physical or emotional stressor. Although its exact pathophysiology is still a matter of debate, a catecholamine surge is thought to play a key role in the genesis of reversible myocardial stunning. Nonetheless, its transient nature, the acute phase of TTS could present serious cardiac complications such as acute heart failure and cardiogenic shock due to systolic dysfunction or to dynamic left ventricular outflow obstruction, and life threating ventricular arrhythmias, with in-hospital mortality of 4–5% (8). The InterTAK diagnostic criteria for TTS have been proposed, and have become a useful tool for the clinician, enabling a bedside diagnosis of this rare condition (9).

As the best of our knowledge this is the first published report of a case of TTS- Afatinib- related. There is no clear explanation for why our patient developed TTS, nevertheless, we may hypothesize that EGFR receptors were severely saturated by Afatinib and covalently bound adducts to plasma proteins, increasing the half-life of this drug that is known to be an ErbB irreversible blocker (10), giving a greater chance of acting on cardiomyocytes-EGFR receptors resulting in a TTS.

This relationship is based on remarkable and highly suggestive findings in the time line sequence:

– Clinical presentation, with a calculated Inter TAK score = 80 (female sex, emotional and physical stress, non-ST segment depression and prolonged QTc time), corresponding to an estimated 97.3% probability of TTSInitial echocardiographic evaluation suggesting typical, apical variant TTS: akinesis of the apical and middle segments, with a circumferential pattern in the GLS plot (apical ballooning).
– Patient‘s clinical recovery in a few weeks.
– Echocardiographic follow-up, with a complete regression of initial findings.
– For debate, the existence of drug related skin toxicity and the possible “EGFR saturation mechanism” of cardiotoxicity raised above.

## Limitations

Despite the high clinical probability supporting TTS, ruling out coronary artery disease is often required for a definitive diagnosis. With coronary angiography ruled out, coronary computed tomography angiography (CCTA) was an excellent alternative to perform a non-invasive evaluation of coronary anatomy in this case, and has been suggested in patients at high risk of complications associated with formal coronary angiography, such as those with advanced oncological disease. Unfortunately in this patient, CCTA was initially not possible due to persistent tachycardia, and was later discarded due to progressive -and finally, complete- recovery of wall motion abnormalities demonstrated by TTE. A cardiac magnetic resonance would have been very useful, because its superior capacity for tissue characterization, being able to differentiate the presence of edema and absence of late gadolinium enhancement, from other fibrosis patterns suggestive of myocarditis or epicardial vasospasm (11).

Regarding the use of strain (GLS) technique as a key tool in the diagnosis of this patient, it is worth mentioning that In situations of acute heart failure, focused ultrasound (focus) is in first line of care (12). Although the usefulness of SGL in multiple pathologies is promising, there is still not enough evidence to justify its use as a first-line or gold standard diagnostic technique, especially in areas of uncertainty and where the technique has not been sufficiently validated to be incorporated into main heart disease clinical practice guidelines. Although its use in detecting cardiotoxicity in oncological patients acquires greater strength day by day, in acute HF (as in this case) its use is also doubtful, especially in the setting of the use of VAD and/or another circulatory assistance (13). Despite all the above limitations, and considering the lack of availability of other techniques, the echocardiogram and especially the use of GLS was a key element in the diagnosis.

An interesting point to comment on, and which could be a subject of debate in the future, will be the assessment of the need and usefulness of an echocardiogram prior to the use of this drug in selected patients. According to the recommendations published by the recent guidelines of the European Society of Cardiology (ESC), the use of echocardiography prior to the start of cancer treatment is reserved for high or very high-risk patients according to baseline stratification (class I-C recommendation). So far, the use of afatinib does not have a strong causal correlation with cardiotoxicity reported in the literature, especially in severe cases such as this one. More evidence is needed to allow, if it were the case, to reclassify this drug as a higher risk of cardiotoxicity, something that has not been consistently observed until now, as compared with other such as anthracyclines or trastuzumab (14).

## Conclusion

So far, afatinib has been seldom associated with cardiovascular toxicity. We report a unique case of afatinib related-TTS in a patient with NSCLC, an association that must be confirmed by future reports. Given the existence of an important pathophysiological basis for cardiac damage, the cardiovascular and oncological communities should be aware of the potential cardiotoxic risk in patients that are under TKI treatments, including afatinib and other TKI that target EGFR.

## Data availability statement

The datasets presented in this article are not readily available because this is a clinical case. Requests to access the datasets should be directed to corresponding author.

## Ethics statement

Written informed consent was not required for this study in accordance with the local legislation and institutional requirements.

## Author contributions

GR and CC: design, writing of the manuscript, revision of the literature, and approval of the article. JB: revision and writing of manuscript and approval of the article. SP: writing of case and approval of the article. MZ: providing images of basal and follow up echocardiograms, revision of manuscript, and approval of the article. AD: revision of the literature and approval of the article. MR: approval of the article. All authors contributed to the article and approved the submitted version.

## Supplementary material

Supplementary Videos 1–3 Transthoracic apical views Echocardiography (Focus) at day 0. Apical view of 4, 2 and 3 chambers with apical and mid akinesia of all segments. Global systolic function is severely impaired.
